# Ultraviolet A irradiation induces ultraweak photon emission with characteristic spectral patterns from biomolecules present in human skin

**DOI:** 10.1038/s41598-020-78884-0

**Published:** 2020-12-10

**Authors:** Katsuhiko Tsuchida, Masaki Kobayashi

**Affiliations:** 1 Shiseido Global Innovation Center, 1-2-11, Takashima, Nishi-Ku, Yokohama, 220-0011 Japan; 2 Graduate Department of Electronics, Tohoku Institute of Technology, Sendai, Japan

**Keywords:** Biophotonics, Skin diseases, Photobiology

## Abstract

Oxidative stress is associated with photoaging of the skin as well as with skin cancer, and is therefore, critical to monitor. Ultraweak photon emission (UPE) is extremely weak light generated during the oxidative process in the living body and has been used as a non-invasive and label-free marker for the evaluation of oxidative stress. However, the mechanism of UPE generation is not clear. Therefore, we aimed to elucidate the molecular mechanism underlying UPE generation by analyzing the spectra of UPE generated from biomolecules in the skin during ultraviolet A (UVA) exposure. The spectra of UVA-induced UPE generated from linoleic acid, linolenic acid, elastin, phospholipids, and 5,6-dihydroxyindole-2-carboxylic acid were measured, and the spectrum of human skin tissue was also obtained. The spectral patterns varied for the different biomolecules and the peaks were distinct from those of the skin tissue. These results suggested that the UPE generated from skin tissue is a collection of light emitted by biomolecules. Moreover, we proposed that UPE is generated through a photosensitization reaction and energy transfer. The identified characteristic spectral patterns of UPE can be useful to elucidate UVA-induced oxidative stress in the skin, with implications for prevention and treatment of photoaging and skin diseases.

## Introduction

Redox reactions continuously occur in the living body to regulate various physiological and cellular processes; among these, the oxidation reaction causes oxidative stress and is associated with various diseases when unregulated^1–3^. Oxidative stress has been recognized as a factor contributing to aging and progression of multiple neurodegenerative diseases^4^. Therefore, it is necessary to elucidate the mechanism of oxidation, for the development of anti-aging strategies and the treatment of related diseases.

The phenomenon of oxidation has also attracted attention in the field of dermatological research, since oxidative stress is associated with skin photoaging and skin diseases^5–7^. Particularly, skin cancer is a serious problem in the dermatological field and is related to excessive levels of oxidative stress^8,9^. Ultraviolet (UV) irradiation is a major generator of oxidative stress. Therefore, it is important to investigate the mechanism of oxidative stress in the skin caused by UV irradiation^10^.

Many methods of evaluating oxidative stress have been developed to date. In vitro, oxidative stress is commonly assessed by measuring the levels of reactive oxygen species (ROS) in cells and of radicals in liquids using specific reagents^11^. In vivo, transgenic mice expressing a redox-sensitive fluorescent protein have been used to assess oxidation in the skin^12^. In addition, oxidative stress can be detected using ultraweak photon emission (UPE) that is spontaneously generated in living organisms^13^. UPE, also referred to as biophoton, is an extremely weak luminescence emitted from the living body, including both animals and plants^14,15^. The human skin also exhibits UPE, which reportedly increases under exposure to UV irradiation^16^. UPE is considered to be derived from electronically excited species formed by ROS-induced lipid peroxidation and protein and nucleic acid oxidation^17^. The oxidation of these biomolecules leads to the formation of high-energy intermediates^18^ and their decomposition generates electronically excited species. It has been reported that singlet oxygen (^1^O_2_) directly emits UPE, and other ROS, such as superoxide anion radical.

(O_2_^·−^), hydrogen peroxide (H_2_O_2_), hydroxyl radical (HO^·^), are indirectly involved in the generation of UPE via the oxidation process and photosensitization reaction^17,19^. The key advantages of using UPE for the evaluation of oxidative stress are that it is a label-free method and allows for non-invasive measurements in vivo.

The usefulness of UPE measurement in assessing oxidative stress has been reported before^20,21^. We previously reported that UPE imaging using a cooled charge-coupled device (CCD) camera is useful for assessing oxidative stress in human skin^22^. In this report, we showed that a UPE imaging system could provide useful images to understand the oxidative stress in the skin induced by UVA and UVB. The UV-induced UPE from the skin decreased by applying antioxidants, showing that UPE derived from oxidative stress. In addition, we have proposed that oxidative stress is correlated with wrinkle formation and porphyrin scores in the skin, highlighting the importance of antioxidation measures in preventing skin damage induced by oxidative stress^23^. The dermal layer has been proposed to be one of the sources of UPE^22,24^, suggesting that the measurement of skin UPE could reflect the degree of oxidative stress throughout the skin, including the epidermal and dermal layers. However, the UPE of biomolecules has not been sufficiently measured to explore the origin of UPE generation in the skin. Knowing the detailed origins of UPE will promote the evaluation of oxidative stress using UPE.

Spectral analysis is one of the main approaches to reveal the characteristics of UPE generation. A recent study showed that *Arabidopsis* subjected to mechanical injury exhibited enhanced photon emission, which led to changes in the spectral pattern^25^. Another study demonstrated a difference in the spectral patterns of UPE from the body surface between human breast cancer-bearing nude mice and healthy control mice^26^. These studies demonstrate that spectral analysis is an essential component of UPE research. However, there has been no detailed investigation regarding the mechanism of UPE generation at the molecular level. Many endogenous fluorophores in the skin show a unique emission wavelength, which can be captured using two-photon microscopy^27^. Two-photon excitation occurs by the simultaneous absorption of two photons at infrared wavelengths, leading to the emission of a fluorescence photon. As the emission wavelength of the fluorescence photon in two-photon microscopy depends on the molecular species, it is expected that different biomolecules exhibit different UPE spectra. Based on this premise, we investigated the detailed emission source of UPE in the skin under exposure to UVA irradiation using polychromatic spectral analysis of relevant biomolecules in the skin and a human skin tissue sample. Components that can be oxidized in the skin were selected as biomolecule samples. Elucidation of the characteristics of UPE generated during UVA irradiation can provide new insights into understanding the mechanisms contributing to skin photoaging and skin diseases, which can in turn offer new targets for treatment and prevention.

## Results

**Figure 1:**
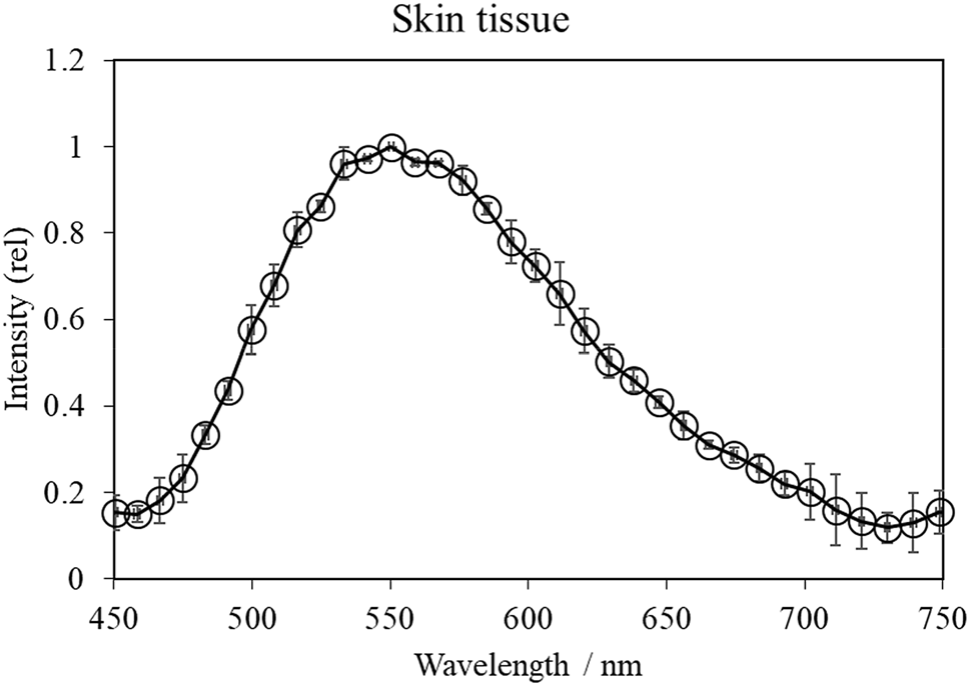
UVA-induced UPE spectrum of the skin tissue. Human skin tissue was irradiated with UVA (1100 mJ/cm^2^) and UPE spectra were measured using a spectroscopy system. The UPE intensity was normalized by the intensity at the peak wavelength and is displayed as relative intensity (rel). Data are presented as means ± SD (n = 3).

**Figure 2:**
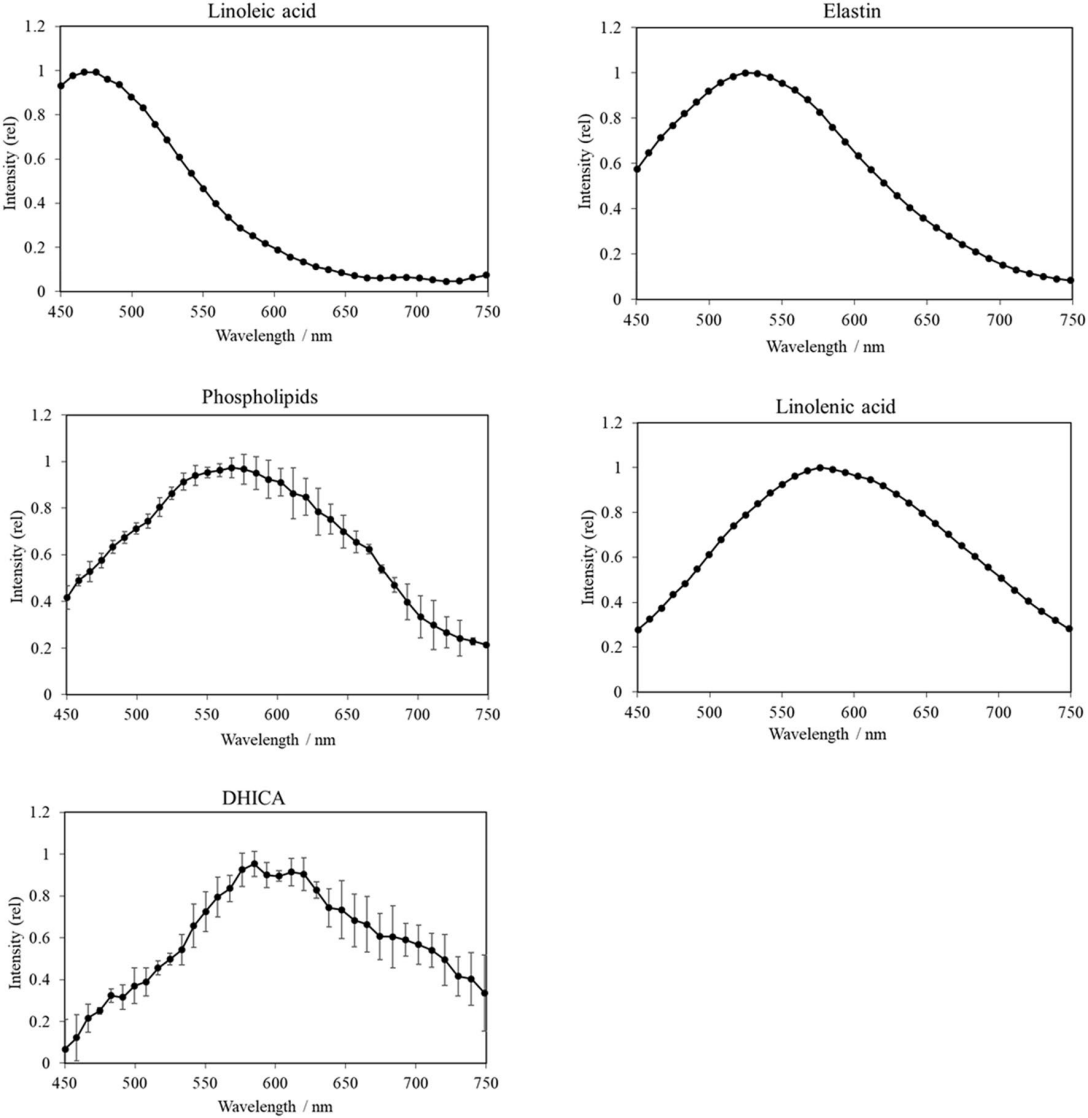
UVA-induced UPE spectra of biomolecules in the skin. Samples were irradiated with UVA (1100 mJ/cm^2^) and UPE spectra were measured using a spectroscopy system. UPE intensities were normalized by the intensity at the peak wavelength and are displayed as the relative intensity (rel). Data are presented as means ± SD (n = 4 phospholipids and DHICA). Spectra of raw materials were measured for all biomolecules except for DHICA, for which a 1 mM solution was used for measurement.

The UPE spectra from five of the seven biomolecules were successfully measured. However, the diluted blood and collagen samples did not exhibit UPE generation induced by UVA irradiation and therefore their spectra were not obtained. UPE intensities at each wavelength were normalized by the intensity at the peak wavelength and the average values were calculated for comparison. The UVA-induced UPE spectra of skin tissue and the individual skin biomolecules are shown in Figs. 1 and 2, respectively, revealing distinct spectral patterns. Specifically, the spectrum for skin tissue peaked at 550 nm, whereas the peaks of linoleic acid, elastin, phospholipids, linolenic acid, and 1 mM 5,6-dihydroxyindole-2-carboxylic acid (DHICA) solution peaked at 475 nm, 525 nm, 570 nm, 575 nm, and 585 nm, respectively. For linoleic acid, the peak was close to the limit of detection (450 nm) of the spectroscopy system on the short wavelength side, suggesting that photons with wavelengths shorter than 450 nm were likely included.

Table 1 shows the wavelength regions for the peak of each sample, which was defined as a relative intensity greater than 0.9. The peak of each biomolecule was within a 40–70 nm range, demonstrating broad peaks similar to the spectrum of the skin tissue. Table 1 also shows the UPE intensities (counts/min) at the spectral peak wavelength. UPE intensity at the spectral peak for the skin tissue was 160 (550 nm), whereas that for linoleic acid, elastin, phospholipids, linolenic acid, and 1 mM DHICA solution was 2760 (475 nm), 10,930 (525 nm), 1230 (570 nm), 5610 (575 nm), and 320 (585 nm), respectively.

**Table 1:** Peak (relative intensity > 0.9) wavelength ranges of UVA-induced UPE spectra and UPE intensities at the spectral peak wavelength.

Emission source	Peak wavelength range (nm)	UPE intensity (counts/min) at peak wavelength
Human skin tissue	530–580	160 ± 11 [550 nm]
Linoleic acid	450–490	2760 [475 nm]
Elastin	500–560	10,930 [525 nm]
Phospholipids	530–600	1230 ± 660 [570 nm]
Linolenic acid	550–620	5610 [575 nm]
DHICA	570–620	320 ± 95 [585 nm]

Data are presented as means ± SD (n = 3 human skin tissue, n = 4 phospholipids and DHICA).

*DHICA* 5,6-dihydroxyindole-2-carboxylic acid.

## Discussion

The oxidation of biomolecules has been suggested to impact several key functions in the living body, including the ones in skin. Therefore, it is important to understand the oxidation potential of each biomolecule for inferring the cause of biological changes and oxidation-related diseases. In the present study, comparison of UVA-induced UPE generation in the entire skin and from individual biomolecules found in the skin that are considered to be strongly affected by oxidation revealed clearly different peaks and wavelength ranges for each biomolecule. The UPE intensity of biomolecules depends on the sample size, and the biomolecular weight used in this study does not match the amount that is actually present in the skin.

A series of lipid oxidation reactions that give rise to various products^28^ may contribute to the spectral range, but since there are further complex contributions, further studies are needed. Linoleic acid (C18:2) has just one less double bond than linolenic acid (C18:2); however, their UPE spectral patterns are remarkably different from each other. This finding suggests that double bonds may majorly contribute to the emission wavelengths of UPE. An in vitro study showed that linoleic acid hydroperoxide, which is formed under oxidative stress, increased the levels of pro-matrix metalloproteinase (MMP)-1 in cultured arterial endothelial cells and increased the levels of both proMMP-1 and proMMP-3 in cultured smooth muscle cells^29^. MMPs are involved in processes such as tissue remodeling and degradation of the extracellular matrix containing collagen and proteoglycans. Similarly, linoleic acid hydroperoxide was shown to significantly increase the production of MMP-1 and MMP-3, suggesting that lipid peroxides are able to alter collagen metabolism^30^. Another study showed that the expression level of MMP-1 increased in fibroblasts after UVA irradiation and sun exposure contributed to the formation of facial wrinkles^31^. Collectively, these findings suggest a relationship between UV light induced lipid peroxidation and wrinkle formation. Indeed, our previous study confirmed a relationship between oxidative stress and wrinkle formation in facial skin^23^. α-Linolenic acid accumulates in the body of mammals (carcass, adipose, and skin) and its major metabolic route is beta-oxidation. A small proportion of ingested α-linolenic acid is converted to docosahexaenoic acid (DHA)^32^, which has been shown to improve skin wound healing in rats^33^. Due to these beneficial effects of DHA produced by the oxidation of linolenic acid, it is relevant to investigate the oxidation of linolenic acid.

The UPE wavelengths obtained in the present study for elastin and phospholipids were similar to their reported fluorescence emission ranges^34,35^. UV-irradiated elastin was previously shown to produce hydrogen peroxide^36^ and oxidation was reported to cause structural changes to elastin^37^. The effects of oxidized phospholipids suggest their potential relevance in different pathologies, including atherosclerosis, acute inflammation, lung injury, and many other conditions^38^. In contrast to elastin and phospholipids, the UPE wavelength region of DHICA, a melanin precursor^39^, was slightly longer than the reported fluorescence range of melanin^27^ and tyrosine^34^, a known DHICA precursor^39^. DHICA oxidized by ^1^O_2_ loses the capacity to induce DNA damage^40^. Considering the fluorescence lifetime^41^, UPE is different from fluorescence emission as it has a longer emission lifetime. For example, immediately after stopping UV irradiation, the UPE lifetime is in the order of minutes (more than 5 min)^21^. Due to this difference in the emission lifetime, it is thought that the emission mechanism of UPE is different from that of fluorescence, wherefore the UPE spectra are also different from fluorescence spectra. Therefore, it is important to examine the unique spectral patterns of UVA-induced UPE.

Considering the emission wavelength of the excited species that cause UPE, the wavelengths of the triplet excited carbonyl are in the range of 350–550 nm^17^. It has been reported earlier that peak wavelength ranges of UPE spectra of biomolecules (DNA and amino acids, such as Cys and Trp) oxidized by hydrogen peroxide and Fe^2+^ are within the wavelength range of the triplet excited carbonyl^42^, however, no such study has been performed for UVA irradiation. In the present study, the peak wavelength range of linoleic acid (450–490 nm) was close to the emission wavelength range of the triplet excited carbonyl (350–550 nm), indicating that triplet excited carbonyls generated by UVA irradiation might have contributed to the spectral pattern of linoleic acid.

We could not obtain the UPE spectra for blood and collagen using our spectroscopy system. For the blood sample, we surmise that hemoglobin absorbed the generated UPE since hemoglobin is known to absorb light in the visible region^43^, which matched the wavelength range of UVA-induced UPE. Our previous study showed no correlation between UPE intensity and the a* value reflecting the cutaneous blood volume^44^; however, photon emission from plasma in blood has been reported to have a UPE spectral range with a peak wavelength of 500–700 nm^45^. In this study, the plasma was not separated from the blood in order to confirm the UPE generation from whole blood. However, blood may indirectly be involved in UPE generation through energy transfer. Although collagen is known to be oxidized^46,47^, we did not detect the photon emission from UVA-irradiated collagen. This result is consistent with the findings of a previous report^24^, suggesting that UVA-induced UPE wavelength of collagen is outside of the detectable wavelength range as inferred from the fluorescence emission wavelength of collagen^27,34,35^.

**Figure 3:**
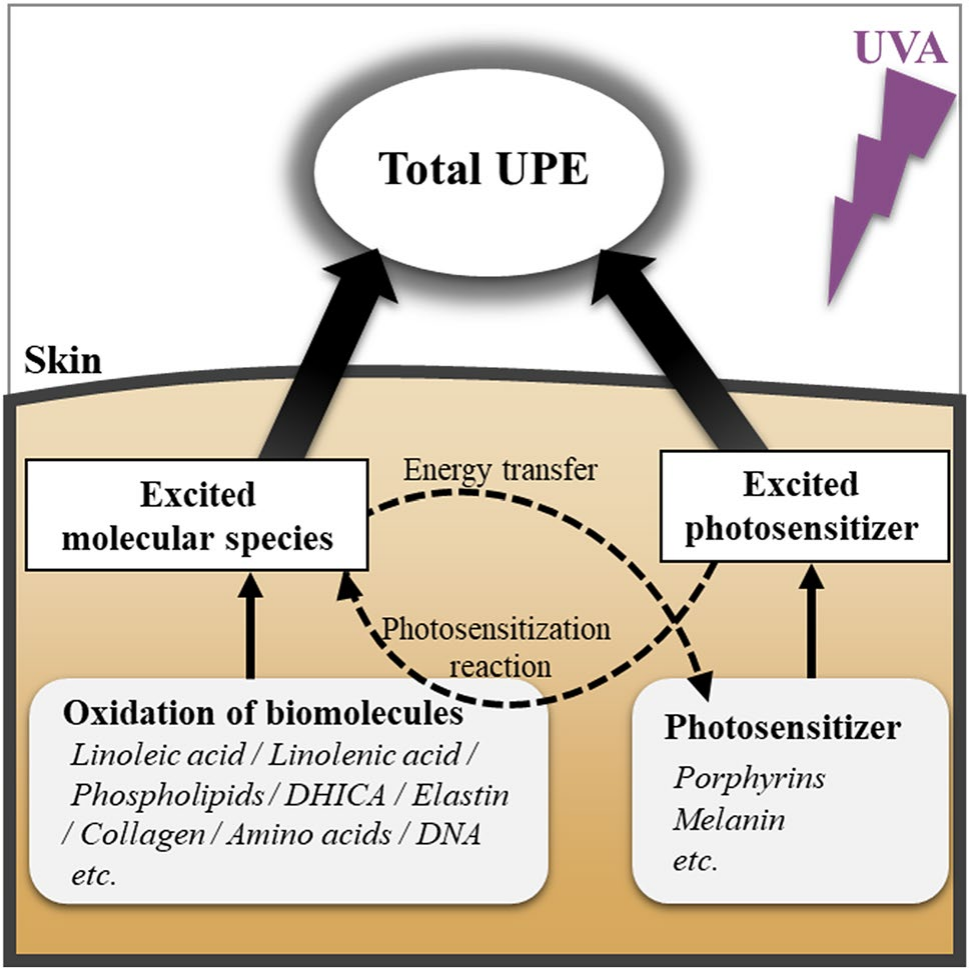
Scheme of the proposed mechanism of UPE generation in the skin by UVA irradiation. UVA irradiation causes UPE generation through oxidation of biomolecules in the skin via several pathways. Biomolecules exposed to UV directly generate UPE, while photosensitizers in the skin are excited by UV exposure. The excited photosensitizers oxidize biomolecules, and UPE is generated via the photosensitization reaction. The excited photosensitizer itself can act as a UPE source too. The UPE measured by the spectroscopy system, therefore, must have comprised the UPE from each of these pathways with a broad spectral pattern that peaked at 530–580 nm.

Based on our present findings and previous reports, we propose some potential pathways for UVA-induced UPE generation in the skin (Fig. 3). UVA directly causes UPE generation through the oxidation of biomolecules in the skin, and then the UPE emission derived from the biomolecules is released from the skin surface. Conversely, it has been suggested that UPE is generated via a photosensitization reaction^48^. The chromophores in human skin act as photosensitizers and UVA induces the excited photosensitizers leading to ROS generation. Porphyrin^49^ and melanin^50^ in the skin are known to cause photosensitization reactions, and other chromophores such as bilirubin, NAD(P)H, *trans*-urocanic acid, and tryptophan have also been proposed as photosensitizers^51^. Our previous study reported a correlation of porphyrin with UPE^23^. UVA excites photosensitizers in the skin, which in turn oxidize biomolecules, thus resulting in the release of UPE from the skin surface. Moreover, the excited photosensitizer itself can act as an UPE source^17^. However, some biomolecules can receive the energy of excited molecular species in the skin, and it is believed that photons of different wavelengths are emitted through energy transfer. In this way, a more complex mechanism of UPE generation and energy transfer may occur in the skin, suggesting that UPE is detected as an assembly of photons of various wavelengths. Although we focused specifically on UVA-induced UPE in this study, a separate mechanism may be operating with respect to UVB-induced UPE since there are chromophores that specifically absorb light in the UVB region^52^.

Although a complex scheme is inferred (as shown in Fig. 3), the results show that the UPE spectrum of phospholipids and the spectrum of skin tissue are similar. Hence, it is proposed that in UVA-exposed skin, phospholipids oxidation is a major contributor of UPE generation. The results in this study regarding phospholipids showed that the UPE generated was from the direct oxidation of the phospholipids by UVA irradiation. However, ROS is generated in actual skin by a photosensitization reaction with UVA irradiation and it is thought that phospholipids are also oxidized by ROS. Phospholipids are the main components of the cell membrane^53^, and therefore, it is important to understand the oxidative stress of phospholipids since their oxidation may have a considerable effect on cell function. However, since other biomolecules are also considered to be contributors of UPE generation from the skin, the wavelengths of UPE emission for each biomolecule confirmed in this study are expected to contribute toward elucidating the complicated mechanism of UPE generation.

Our study had some limitations that should be taken into consideration when evaluating the results. It is difficult to elucidate the mechanism underlying UPE generation in response to UVA irradiation from the obtained data. As the skin is composed of various components it was not feasible to evaluate all the biomolecules, therefore, we focused on the major biomolecules known to oxidize in the skin. Herein, the mechanism of UPE was discussed with respect to the previously reported spectral analysis of biomolecules as well as the studies on UPE; however, further research involving other skin biomolecules is required. Nonetheless, we believe that the data presented contribute to the elucidation of the mechanism involved in UPE generation. In turn, it is expected to provide insights into the mechanisms underlying skin photoaging and development of skin diseases in response to UV exposure.

## Methods

### Skin tissue samples

A human skin sample from the breast of a 58-year-old Caucasian female was purchased from Biopredic International (Rennes, France) via KAC (Kyoto, Japan) and stored frozen. The donor provided written informed consent for the use of the skin sample. Biopredic International and KAC comply with ethical standards for research involving human samples. The subcutaneous tissue was physically removed from the skin sample prior to UVA irradiation and spectral measurements. Three smaller pieces of skin tissue were prepared from the skin sample. Subsequently, all the three tissue pieces were, independently, irradiated with UVA, and the UPE spectra were measured. The skin tissue pieces that were UVA-irradiated once and used for spectrum measurement were not used again in the experiment.

### Skin biomolecule assessment

The following seven biomolecules were selected for spectral analysis: phospholipids (solid, non-hydrogenated egg phosphatidylcholine; Coatsome NC-50, NOF Corporation, Tokyo, Japan), elastin (solid, from bovine neck ligament powder; E1625, Sigma-Aldrich, St. Louis, MO, USA), linoleic acid (liquid, α-linolenic acid; 126-03612, Fujifilm Wako Pure Chemical Corporation, Osaka, Japan), linolenic acid (liquid, 20526-52, Nacalai Tesque, Kyoto, Japan), collagen (solid, VitroCol lyophilized type I collagen from human; 5008, Advanced BioMatrix, Inc., San Diego, CA, USA), DHICA (solid, Tokyo Chemical Industry Co., Ltd., Tokyo, Japan), and whole blood (liquid, human heparin sodium whole blood; BioIVT, West Sussex, UK via KAC, Kyoto, Japan). For measurement, 1 mM DHICA was dissolved in 0.1 M phosphate buffer (pH 7.4), the whole blood was diluted tenfold with Dulbecco's phosphate-buffered saline (−), and raw samples were used for the other molecules.

### UVA irradiation

UVA irradiation was generated using a Dermaray 200 system (Canon Medical Supply, Tokyo, Japan) with a UVA source (TOREX FL20SBL/DMR, 300–430 nm, peak 352 nm; Toshiba Medical Supply, Tokyo, Japan). The UVA intensity was measured by DMR-UV-ABBNB-1 (Gigahertz-Optik GmbH, Puchheim, Germany). The skin tissue and each of the biomolecule samples were irradiated with UVA at 1100 mJ/cm^2^, 2.3 mW/cm^2^.

### Spectroscopy

**Figure 4:**
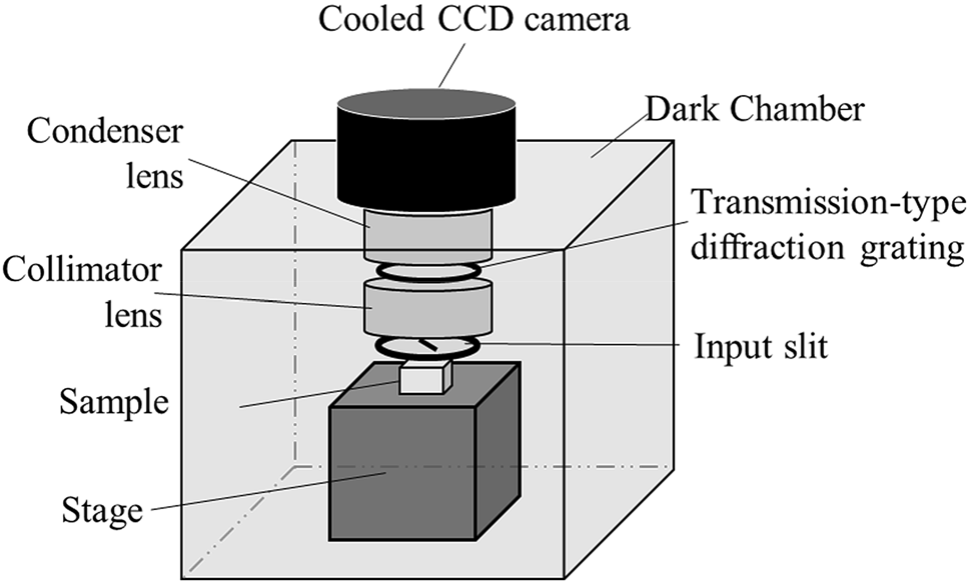
Schematic illustration of the polychromatic spectroscopy system. The UPE spectra of the samples were measured using a polychromatic spectrum analysis system, which consists of a transmission-type diffraction grating, condenser lens, collimator lens, input slit, and cooled CCD camera. The samples were irradiated with UVA outside of the dark chamber and were then immediately placed under a 1 mm wide and 20 mm high optical input slit in the dark chamber of the spectroscopic system.

The UPE spectra of the human skin tissue and biomolecule samples were measured using a polychromatic spectrum analysis system developed by Kobayashi et al.^54^ (Fig. 4), which consists of a transmission-type diffraction grating, condenser lens, collimator lens, input slit, and cooled CCD camera (SI 600 s; Spectral Instruments Inc., USA). The detection wavelengths of this spectroscopy were in the range of 450–750 nm. Unlike spectroscopic devices that continuously detect UPE at each wavelength, the spectroscopic system used in this research detected UPE at each wavelength at the same time. Thus, there was no lag time in the detection of each wavelength.

The measurement procedure was as follows: The sample was irradiated with UVA outside the dark chamber. After UVA irradiation, the liquid biomolecule samples were immediately placed in a borosilicate glass cuvette, which was placed under a 1 mm wide and 20 mm high optical input slit in the dark chamber of the spectroscopic system. In contrast, solid biomolecule samples and the skin tissue were immediately set on the plate after UVA irradiation and placed directly under the input slit in the dark chamber. The skin tissue was placed with the stratum corneum facing the input slit. Immediately after UVA irradiation and sample placement, the spectrum was recorded. Exposure times for spectral measurement of UV-induced UPE were set to 20 min for the skin tissue and 5 min for the biomolecule samples. UPE intensities at each wavelength were normalized by the intensity at the peak wavelength and are expressed as the relative intensity to facilitate direct comparisons. Spectra of the skin tissue and biomolecule samples were measured several times depending on the emission intensity to confirm reproducibility. Specifically, the skin tissues’ spectra were measured three times, while those of linoleic acid, elastin, and linolenic acid were measured twice each, and for phospholipids and DHICA they were measured four times each.

### Experimental protocols statement

All experimental protocols were approved by Shiseido Co. Ltd., and Tohoku Institute of Technology.

## Data Availability

The datasets generated and/or analyzed during the current study are available from the corresponding author on reasonable request.
